# Pros and cons of the application of evolutionary theories to the evolution of SARS-CoV-2

**DOI:** 10.2217/fvl-2020-0048

**Published:** 2020-04-17

**Authors:** Yue Li, Xinai Yang, Na Wang, Haiyan Wang, Bin Yin, Xiaoping Yang, Wenqing Jiang

**Affiliations:** 1 Department of Respiratory Diseases, Qingdao Haici Hospital, Qingdao, Shandong, PR China

**Keywords:** application, concerns, evolution and origin, RNA virus, SARS-CoV-2

## Abstract

The outbreak of severe acute respiratory syndrome coronavirus 2 (SARS-CoV-2) has caused severe damage to the world. With the support of classic evolutionary theories and population genetics principles, many studies on the origin of SARS-CoV-2 have revealed encouraging results but meanwhile some are still under debate. We are concerned with the validity of applying classic evolutionary theories and formula to the evolution of RNA viruses. We have raised several confounding factors like the RNA replication feature and the RNA modification systems of the hosts, which might jeopardize the validity of the application of classic methods to analyze the SARS-CoV-2 data.

The outbreak of severe acute respiratory syndrome coronavirus 2 (SARS-CoV-2) has caused severe damage to China, especially the Hubei province [1–3]. Recently, the cases in China are coming under control but the situation in some other countries has become exacerbated. It is urgent to find ways to control its transmission and cure the infected patients.

The emergence of papers on the evolution of SARS-CoV-2 is as fast as the spread of the virus itself. Based on genome sequencing followed by sequence alignment and sequence similarity analysis, researchers have characterized the evolutionary patterns of SARS-CoV-2 and postulated its origins. Theories of bat origin [4–6] and pangolin origin [7,8] emerged and even the snake plays an intermediate role in the transmission of SARS-CoV-2 [9]. Many other similar studies and results are not exhaustively listed here at all. In a word, it seems that the continuous change of theories on the evolution of SARS-CoV-2 even exceeds the evolution rate of the virus itself. It seems that several results appear to be encouraging. For example, a recent paper by Tang *et al*. [10] has taken advantage of RNA-seq data. By traditional mutation calling pipeline, the authors discovered two lineages (L and S) of SARS-CoV-2, and claimed that the L type is more virulent than S type based on their frequency among the population. This might be a typical bioinformatic study that informs us of the scenario of SARS-CoV-2 population and evolution. The ‘snake’ paper [9] also emphasized the important fact that viruses adapt to the host expression system, so that their codon content should adapt to the host environment. No matter whether the final conclusion is correct or not, the main contribution is that the associated terminologies like relative synonymous codon usage and codon adaptation index are nicely incorporated in the relevant studies, indicating a correlation between the codon content of host and parasite.

As far as we know, the classic theories and principles of evolutionary biology, especially population genetics, are based on the central dogma, where DNA should be first transcribed to RNA and then produce proteins, and meanwhile the DNA replicates itself under a certain error rate. The validity of many formula/software is also based on multiple hidden hypotheses such as random mating, bi-allelic DNA, bi-sexual gender and infinite population size.

However, SARS-CoV-2 is a positive-strand RNA virus. It reproduces its own RNA by RNA replication. We doubt the validity of applying classic evolutionary theories and formula to the evolution of RNA viruses. In this article, we would raise and discuss several concerns regarding how the confounding factors would jeopardize the validity of the application of classic methods to analyze the SARS-CoV-2 data. We should emphasize that we neither criticize any studies, nor prove any ideas wrong. Instead, we aim to raise some questions and wish that these concerns could be further discussed by the broad community. We hope our concerns would contribute to the accurate identification of the origins of SARS-CoV-2.

## The problem of RNA modification system in host cells

We have stated that SARS-CoV-2 is an RNA virus. The classic theories, principles and formula of evolutionary biology are based on the central dogma, which assumes the DNA-to-RNA-to-protein chain. The basic notion is that the mutations on DNA could be inherited rather than the modifications on RNA. For example, despite the numerous types of RNA modifications in higher eukaryotes, these organisms do not suffer from extraordinarily high mutation rates because the modified RNAs could not be transmitted to the next generation. However, for RNA viruses, their RNA is actually their genetic information. Whether the evolutionary principles could be applied to RNA viruses should be seriously debated. The host cells have multiple RNA modification systems/enzymes. The changes in viral RNA (by host cells) would permanently change its genetic information and be transmitted to the next ‘generation’, which is similar to genetic mutations in higher organisms. Technically, one could not distinguish genetic mutations and RNA modifications from the RNA-sequencing data of RNA viruses. So, what is the point of detecting positive/negative selection based on the mutations in the virus population? These mutations are possibly conferred by the host cell’s RNA modification systems. How could the randomly occurring RNA modification events have preference on missense or synonymous sites?

The recent study by Tang *et al.* [10] claimed that the divergence between SARS-CoV-2 and RaTG13 (a bat SARS-related coronavirus) is 14-times larger than the divergence between human and chimpanzee. The authors concluded that only the neutral evolving sites should be considered rather than all different sites. Let us assume that both SARS-CoV-2 and RaTG13 undergo the RNA modification by host cells, and the modified viral RNA is inheritable, then their sequence (SARS-CoV-2 and RaTG13) could become quite different within a short time scale. The divergence time is calculated as t = dS/2u. When dS might be largely contributed by the RNA modification system of host cells, this estimation could be inaccurate. In the dN and dS calculation, it is necessary to rule out any mismatch sites that might be produced by RNA modification. They should at least mention why they should or should not consider this factor. And then, the mutation rate ‘u’, how to define ‘u’? Does ‘u’ include the nucleotide changes conferred by the host’s modification enzymes? Therefore, the authors' logic chain is questionable.

More importantly, the single nucleotide polymorphism and modified RNAs are technically indistinguishable. The software and algorithms only align the sequences but do not tell you whether the observed mismatch is a single nucleotide polymorphism or RNA modification site. This is a biological problem rather than technical problem, and could not be solved by adjusting or improving the alignment parameters or filtering criteria.

One may argue that some studies have analyzed RNA modifications by using classic evolutionary theory, but note that the viral RNA is modified by the host cell rather than by the virus itself, and their equivalence (modified by the host or modified by itself) should be formally proven before conducting any analyses.

Indeed, the coronaviruses isolated from the hosts (like human, bat or pangolin) are only compared with SARS-CoV-2. Therefore, RNA virus sequence is compared with the SARS2 RNA, and hence, the used strategy is almost sound. So, these studies compared virus to virus or RNA to RNA and conclude the potential host or carrier based on the highest percent of identity or potential concluded recombination events. However, as we have stated, the sequence similarity could be largely and even randomly skewed by the hosts' RNA modification systems. Therefore, aligning RNA with RNA is fine, but the concern is how to distinguish whether the observed divergence (or mismatch sites) really reflects the phylogeny of the viruses. It could simply be shaped by the arbitrary modification of the host’s enzymes.

The next concern caused by RNA modification system is the ‘batch effect’. In population genetics, DNA mutations take place randomly among different individuals. But when RNA viruses are modified by host cells, the modification enzymes are likely to modify multiple sites at a time. Since the genes of an RNA virus (like the 12 genes of SARS-CoV-2) are linked, they are prone to be modified ‘in a batch’. In contrast, in higher organisms, it is not possible to see numerous individual-specific mutations linked within an entire haplotype unless it is driven by selective sweep. This reality again challenges the application of traditional theories to RNA viruses.

In our opinion, the RNA viruses should obey a different evolutionary theory. So far, the functional experiments are more important and reliable than the pure evolutionary analyses in this case of RNA virus. When traditional evolutionary principles are jeopardized by additional mutation forces, the functional experiments work well as they did in the past.

## Problems raised from the RNA replication process

Apart from being modified by the host RNA modification systems, there are other concerns about whether the evolutionary theories could be applied to the RNA viruses like SARS-CoV-2.

First, for cellular organisms, the DNA mutations are majorly introduced during the DNA replication process. The mutation rate is largely connected with the fidelity of DNA replication. The next step is the natural selection force acting on these mutations, after which the deleterious mutations are purged and those beneficial mutations are maintained. However, RNA viruses either undergo the reverse-transcription process (like HIV) or the RNA replication process (like SARS-CoV-2). For RNA viruses, every newly transcribed RNA molecule is a potential offspring of the original virus. The mismatches introduced during reverse transcription or RNA replication would be maintained and kept in the offspring. Before applying the evolutionary formula to RNA viruses, one should state whether RNA replication has similar mismatching rates as DNA replication. Intuitively, DNA–DNA pairing (DNA replication), DNA–RNA pairing (transcription) and RNA–RNA pairing (such as RNA replication) should have different mismatching rates. Thus, when applying theories to SARS-CoV-2, should the authors consider the potentially different mutation rates during the reverse transcription or RNA replication processes? Take the paper by Tang *et al.* [10] for instance, what exactly does mutation rate ‘u’ refer to? Even the problem of technically indistinguishable RNA modification and the ‘*de novo’* RNA mutation is not mentioned by the authors at all, let alone the mutations introduced during the RNA replication process. At least, the authors could briefly introduce the reproduction mode of SARS-CoV-2 rather than ‘mechanically’ apply the formula to an organism which they are not familiar with.

Second, the DNA generated from the reversetranscription is only an intermediate, and could not be packaged into the envelope protein. The same goes for the negative-strand RNA generated by the positive-strand RNA during the RNA replication. So that the DNA/negative-strand RNA and any changes on it could not be directly transferred to the next host cell. However, if a DNA intermediate could transcribe *N* RNA molecules, and a mutation takes place when *N*/2 RNA molecules have been transcribed, then only part of the offspring would have this mutation. All these random processes could not be predicted by any algorithms. How could theories on DNA mutations be applied to a transiently existing DNA intermediate or negative-strand RNA?

These factors may shed concerns on the accuracy and validity of the results of previous works. However, we do not claim any studies to be wrong since we have neither better methods nor enough evidence. Hopefully, our opinions could be seen and discussed by other researchers and benefit the studies on the evolution and origin of SARS-CoV-2.

## Conclusion

We are concerned with the validity of applying classic evolutionary theories and formula to the evolution of RNA viruses. We have raised several confounding factors like the RNA replication process and the RNA modification systems of the hosts, which might jeopardize the validity of the application of classic methods to analyze the SARS-CoV-2 data. However, we neither criticize any studies nor prove any ideas wrong. We hope our concerns could be considered by the broad community and could contribute to the accurate identification of the origins of SARS-CoV-2.

## Future perspective

We anticipate and speculate that the field of virus evolution could be incorporated with more novel evolutionary theories that distinguish cellular organisms, DNA viruses and RNA viruses, due to their distinct features of nucleotide components, mutation rates, and reproductive patterns.

**Executive summary**

- The recent evolutionary studies on SARS-CoV-2 have revealed encouraging results as well as debatable questions.
- The problem of RNA modification system in host cells: RNA modification system in the host cells could act on viral RNAs, which jeopardizes the validity of many hidden hypotheses behind the evolutionary theories.
- Problems raised from the RNA replication process: the traditional evolutionary theories did not mention the RNA replication process at all, while SARS-CoV-2 exactly uses this approach to replicate itself.
